# Lanthanide-Doped REVO_4_ (RE = Y, Gd, Lu, La) Phosphors: From Synthesis to Sensing Applications

**DOI:** 10.3390/s26092660

**Published:** 2026-04-24

**Authors:** Dragana Marinković, Giancarlo C. Righini, Maurizio Ferrari

**Affiliations:** 1 Vinča Institute of Nuclear Sciences, National Institute of the Republic of Serbia, University of Belgrade, P.O. Box 522, 11001 Belgrade, Serbia; 2 Nello Carrara Institute of Applied Physics (IFAC CNR), Sesto Fiorentino, 50019 Firenze, Italy; righini@ifac.cnr.it; 3 Institute of Photonics and Nanotechnologies (IFN CNR, CSMFO Laboratory) and FBK Photonics Unit, Via alla Cascata 56/C, Povo, 38123 Trento, Italy

**Keywords:** lanthanide, rare-earth, vanadate, synthesis, luminescence, downconversion, upconversion, sensing applications

## Abstract

Rare-earth elements including the fifteen lanthanides, from lanthanum (La) to lutetium (Lu), together with scandium (Sc) and yttrium (Y), can act either as matrix cations or as active luminescent centers when incorporated into host lattices. Owing to their relatively large ionic radii, high coordination numbers, and structural stability, ions such as La, Lu, Sc, Y, and gadolinium (Gd) typically serve as matrix cations in rare-earth vanadate (REVO_4_)-based phosphors, while other trivalent lanthanide (Ln^3+^) ions act as active luminescent centers. These REVO_4_ phosphors have proved to be good host lattices for optically active Ln^3+^ ions giving strong luminescence assigned to absorption of the vanadate (VO_4_^3−^) groups, and the efficient energy transfer between host lattice and Ln^3+^ ions. The unique electronic configuration of Ln^3+^ ions, particularly their unpaired 4f electrons, makes them ideal for applications in luminescence, magnetism, electronic and magnetic relaxation, and catalysis. Due to their complementary luminescent characteristics, Ln^3+^-doped REVO_4_ phosphors have attracted significant attention in recent years. Their unique optical properties make them highly valuable across a broad spectrum of applications. This paper provides a comprehensive review of the state of the art in Ln^3+^ (Eu^3+^, Sm^3+^, Tm^3+^, Er^3+^, Ho^3+^, Tb^3+^, Nd^3+^, and Yb^3+^)-doped REVO_4_ (RE = Y, Gd, Lu, La) phosphors. It examines current synthesis approaches, alongside the development of advanced strategies, and explores structural characteristics, innovative designs, and luminescent behavior, including both downconversion and upconversion processes and sensing applications, of the Ln^3+^-doped REVO_4_ phosphors.

## 1. Introduction

Vanadates represent a family of inorganic compounds containing a vanadium ion in its highest oxidation state (+5). The most basic member of this group is the tetrahedral orthovanadate VO_4_^3−^ anion, commonly encountered in sodium orthovanadate or in alkaline solutions of V_2_O_5_ at pH values above 13 [1]. Based on the types of combined cations with VO_4_^3−^ anions, the vanadates can be classified as alkali metal vanadates (M_3_VO_4_) (M = (Li, Na, K, Rb), alkali earth metal vanadates (MV_2_O_6_) (M = Ca, Sr, Ba), transition metal vanadates (MxVyOz), (M = Co, Zn, Ni, Cu, Fe), orthovanadates (MVO_4_) (M = Ln = from La to Lu, Sc, and Y), and other vanadates with NH_4_^+^, Al^3+^, In^3+^, and Bi^3+^ cations. REVO_4_, along with structurally related inorganic compounds such as fluorides, oxides, phosphates, molybdates, tungstates and analogous materials, constitutes an expanding domain of contemporary research. Within the current scientific landscape, numerous research groups have studied REVO_4_ compounds with their crystallographic, electronic and optical characteristics, as well as potential for advanced applications [2,3,4].

REVO_4_ materials serve as versatile hosts for incorporating optically active different Ln^3+^ ions which share the same +3 oxidation state and exhibit close similarities in ionic size, electronegativity, and electronic configuration with the cations of the lattice, so that they can be introduced across a broad concentration range. This substitution occurs without causing significant distortions or alterations in the crystal structure [5].

In addition, incorporation of multiple optically active dopants into the REVO_4_ matrix is essential for enhancing both the performance and versatility of the matrix. By incorporating various co-dopants, researchers are exploring their potential as sensitizers and their ability to expand the functional properties of the medium. These investigations aim to achieve advanced effects such as self-Q-switching, self-mode-locking, self-frequency-doubling, and self-Raman conversion, thereby broadening the operational capabilities of the system [6]. REVO_4_ doped with optically active Ln^3+^ ions is known for its strong luminescent behavior, which arises from efficient energy transfer processes between the vanadate groups and the Ln^3+^ ions. By carefully choosing the type and concentration of these dopants, a broad spectrum of emission colors can be achieved. Both undoped and Ln^3+^-doped REVO_4_ phosphors have been widely investigated as multifunctional materials. Their versatility makes them highly appealing for diverse applications, including use in phosphors, specialty glasses, optical polarizers, and optoelectronic devices. They also play important roles in telecommunications, scintillation detectors, and photocatalysis. Furthermore, REVO_4_ compounds show promise as advanced sensing nanoprobes, offering dual functionality for optical bioimaging and magnetic resonance imaging (MRI) [7,8].

Vanadate-based phosphor compounds have attracted significant interest due to their dual functionality as hosts for Ln^3+^ dopants and as self-activating phosphors. Among them, REVO_4_ materials such as yttrium vanadate (YVO_4_), gadolinium vanadate (GdVO_4_), and lutetium vanadate (LuVO_4_) are particularly interesting. Their strong luminescence, long fluorescence lifetimes, low lasing thresholds, large emission cross sections, and high absorption coefficients have made them valuable for a wide range of optical applications [9,10]. Recent review articles have highlighted the role of self-activated hosts, such as vanadates and niobates, in enhancing and tailoring the optical properties of Ln^3+^ ion dopants, driving significant progress in their synthesis and optimization. Parallel advances in host selection, principally tungstates, vanadates, and aluminates, improved luminescent efficiency and broadened optoelectronic applications. Moreover, vanadate systems have provided insights into catalytic processes relevant to hydrogen and oxygen evolution reactions [11,12,13]. A recently published book chapter [14] examined orthovanadates of mono-, di-, and trivalent metals, focusing on their synthesis, structural information of room-temperature phases, morphology, luminescent properties and potential applications. A literature survey of hydrothermal syntheses of bismuth vanadate was particularly noteworthy. While this book chapter [14] examined photoluminescence in a broad range of orthovanadates, including those with s- and p-block cations, the present review focuses specifically on Ln^3+^-doped REVO_4_ (RE = Y, Gd, Lu, La) compounds and extends the discussion to recent advances in sensing applications.

The improved sensitivity, selectivity, stability, and biocompatibility of Ln^3+^-activated vanadates and the catalytic properties of CeVO_4_ nanoparticles make the vanadate system an excellent candidate in biosensing platforms, cancer therapy, antibacterial activity, biosensing, and drug delivery. These developments place REVO_4_ compounds at the forefront of multifunctional material research. New studies concerning co-doping, nanostructuring, and host lattice engineering will advance biomedical applications and next-generation dosimeter technologies [15,16].

The YVO_4_ material stands out among REVO_4_ compounds as one of the most extensively studied laser materials over the past few decades. Its unique physical characteristics make it highly valuable in diverse applications, ranging from polarizers and gas sensors to phosphors and advanced laser systems, particularly when doped with Ln^3+^ ions [17]. Also, YVO_4_ exhibits remarkable chemical stability along with a broad optical transparency range from 400 to 5000 nm. Owing to these properties, it has found extensive use in optical communication systems and is a key material in the development of light isolators and circulators [18]. Ln^3+^-doped LuVO_4_ materials demonstrate exceptional laser performance, attributed to their stronger absorption at pump wavelengths and enhanced emission cross-sections compared to other orthovanadates. In particular, neodymium-doped LuVO_4_ (Nd:LuVO_4_), which crystallizes in the zircon structure, offers a significantly larger emission cross-section than Nd:YVO_4_ and Nd:GdVO_4_, while maintaining a high threshold for optical damage [19].

On the other hand, GdVO_4_, with its exceptionally high melting point at ~1800 °C, offers several advantages over YVO_4_. It delivers greater brightness, responds efficiently to ultraviolet excitation, and facilitates effective charge-transfer processes. In addition, GdVO_4_ exhibits lower phonon energy and reduced sensitivity to moisture, and can serve in multiple applications either in its undoped form or when doped with optically active Ln^3+^ ions [20,21].

Materials based on Gd^3+^ (4f^7^) ions serve as excellent lattice hosts for developing up-conversion systems, largely because their lowest excited states lie at relatively high energies. The luminescence properties of GdVO_4_ depend on the specific dopant ions incorporated into matrix. When the vanadate is activated by single ions, such as Eu^3+^, Dy^3+^, or Sm^3+^, the material operates as a downconversion phosphor, emitting photons at characteristic wavelengths in the visible region. Conversely, activation by pairs or multiple ions, such as Er^3+^/Yb^3+^, Ho^3+^/Yb^3+^, Tm^3+^/Yb^3+^, or Er^3+^/Tm^3+^/Ho^3+^/Yb^3+^, induces upconversion processes that generate emission at higher energy in respect to the excitation [22]. Furthermore, Nd^3+^ doping expands its luminescent potential, adding another dimension to its versatility. Nd^3+^-doped REVO_4_ compounds combine strong mechanical stability with excellent optical performance, particularly through their pronounced absorption at 808 nm. This wavelength is highly relevant for biomedical technologies, making such materials especially attractive in that field. In addition, nanoscale phosphors derived from GdVO_4_ and co-doped with Nd^3+^ and Yb^3+^ can act as colloidal donor–acceptor systems. These nanophosphors provide a useful platform for investigating interparticle energy transfer phenomena in aqueous nanofluids [23]. Also, Gd-based materials can be used in osteogenic, antimicrobial, and anticancer applications, and in bioimaging and bioprobes. This functionality arises from the presence of unpaired electrons in Gd^3+^ ions, which effectively alter the relaxation dynamics of nearby water protons, thereby enhancing image contrast [24].

The main objective of this article is to review recent literature on undoped and Ln^3+^ (Eu^3+^, Sm^3+^, Tm^3+^, Er^3+^, Ho^3+^, Tb^3+^, Nd^3+^, and Yb^3+^)-doped REVO_4_ (RE = Y, Gd, Lu, La) phosphors, highlighting the current state of the art in synthesis approaches alongside the development of advanced strategies, and exploring structural characteristics, innovative designs, luminescent behavior, including both downconversion and upconversion processes, and sensing applications of the Ln^3+^-doped REVO_4_ phosphors. This review article focuses on the fundamental aspects and advantages of the most widely used synthesis methods, such as solid-state reactions, coprecipitation, hydrothermal/solvothermal routes, sol–gel processes, and microwave-assisted techniques, emphasizing their ability to control particle size, tailor morphology, and optimize dopant distribution to enhance luminescence performance of REVO_4_. The incorporation of optically active Ln^3+^ ions into the REVO_4_ matrix has been shown to influence structural modifications, lattice distortions, and defect center formation, all of which significantly influence emission intensity and stability. Owing to their sharp, well-defined emission lines from f–f transitions from Ln^3+^ ions, combined with strong ultraviolet absorption by VO_4_^3−^ groups, REVO_4_ materials demonstrate excellent photostability, long luminescence lifetimes, and tunable emission colors under both downconversion and upconversion processes. These unique optical properties have enabled their application across a broad spectrum of technological areas. This review highlights applications of the Ln^3+^-doped REVO_4_ phosphors in LED diodes, luminescent electromagnetic displays, optical sensing, lasers, luminescence thermometry, chemical and bio-sensing, biodetection, infrared fluorescence bioimaging, photothermal therapy, photocatalysis, gas sensing, antimicrobial activity, and anti-counterfeiting technologies.

## 2. Synthesis Approaches in the Fabrication of Vanadate-Based Materials

The fabrication of vanadate-based materials with high purity, crystallinity, and well-defined uniform size, morphology, and composition, along with a homogeneous distribution of impurities, is crucial for the advancement of modern functional materials. To be practical, synthetic methods must also be economical, scalable to industrial production, and capable of delivering high yields. These requirements drive the development of novel or modified synthesis procedures aimed at enhancing the characteristics and performance of different kinds of vanadate materials.

In recent years, the design, synthesis, and fabrication of multifunctional materials have gained significant attention. A variety of synthetic approaches can be employed to produce REVO_4_ with diverse sizes, shapes, properties, and morphologies, depending on the intended application. Control over nanoparticle size and shape is typically achieved through careful optimization of reaction parameters. Furthermore, synthesis methods can be classified based on the phase in which the phosphors are formed, i.e., solid, liquid, or gaseous, providing different pathways to tailor their structural and functional properties.

### 2.1. Solid-State Reaction

Inorganic solids are often synthesized by the ceramic method, which involves mixing stoichiometric amounts of solid precursors, grinding them to fine particles, and heating for several hours. For example, the simplest reaction pathway yields GdVO_4_ by combining gadolinium oxide (Gd_2_O_3_) with vanadium pentoxide (V_2_O_5_):

Gd_2_O_3_ + V_2_O_5_ → 2GdVO_4_

This process requires high temperatures to enable ion diffusion, but the large difference in melting points between V_2_O_5_ (681 °C) and Gd_2_O_3_ (2339 °C) complicates synthesis. While diffusion can be aided by fine grain size and mixing, elevated temperatures remain essential. A challenge arises from V_2_O_5_ volatility and decomposition at high temperatures, which can lead to oxygen loss and formation of VO_2_. Literature reports vary: some note incomplete reactions with stoichiometric mixtures, while others suggest adding excess V_2_O_5_ or carefully controlling heating ramps to avoid evaporation. For example, slow heating to 950 °C followed by higher temperatures can stabilize the reaction. To address these limitations, precursors must be thoroughly mixed to maximize surface contact. In some cases, reactions are carried out under vacuum or in an inert atmosphere, which can further accelerate the process. Ultimately, thorough characterization of the product is necessary, as impurities such as unreacted Gd_2_O_3_, GdVO_3_, or non-stoichiometric GdVO_4_ may form due to volatility and reduction of V_2_O_5_ [25,26].

For example, REVO_4_ (RE = Y, Sm, Gd, Yb, Lu) and GdVO_4_:Ho^3+^/Yb^3+^ materials are typically synthesized in the solid phase using a high-temperature solid-state method. In this process, stoichiometric amounts of precursors are thoroughly ground, mixed, and heated in multiple steps at approximately 1200 °C for several hours to ensure complete reaction and formation of the desired product [27,28].

### 2.2. Coprecipitation Method

The coprecipitation method is a simple and effective technique for synthesizing vanadate-based materials with uniform size distribution. The process involves dissolving precursor salts (nitrates, sulfates, chlorides, etc.) in water and adding a precipitating agent (hydroxide, carbonate, or hydrogen carbonate), followed by aging, separating the precipitate, and finally washing and drying. The precipitation rate depends on the specific metal ions used. Huignard et al. demonstrated that Eu-doped YVO_4_ materials can be synthesized at room temperature by coprecipitation from soluble nitrates and sodium orthovanadate. Although their work focused on nanoparticles, the findings aside from size control apply to bulk materials. The reaction is highly pH-dependent, with yttrium orthovanadate forming only within a narrow range (12.5–13.0). At pH values above 13, hydroxides precipitate without reacting, while acidic conditions favor condensed vanadates, preventing orthovanadate formation [29].

The reaction involves, in the first step, the precipitation of the kinetically favored hydroxide:

(Y_x_Eu_1−x_)^3+^ + 3OH^−^ −→ Y_x_Eu_1−x_(OH)_3_ ↓

which, in the second step, reacts with orthovanadate to form thermodynamically stable Y_x_Eu_1−x_VO_4_:

Y_x_Eu_1−x_(OH)_3_ + VO_4_^3−^ −→ Y_x_Eu_1−x_VO_4_ + 3OH^−^

Subsequent studies suggest that similar conditions apply to Gd^3+^ systems, as RE hydroxides are generally insoluble in water but soluble in acids. For bulk reference materials, precise particle size control is unnecessary, and solution synthesis offers the advantage of ambient temperature processing, avoiding issues linked to high-temperature solid-state methods. However, rapid precipitation often produces defective crystalline regions. Thermal treatment has been shown to improve crystallinity, making a two-step process, coprecipitation followed by heating at different temperatures, a viable alternative to solid-state synthesis, while avoiding complications from V_2_O_5_ volatility [22].

### 2.3. Hydrothermal/Solvothermal Method

Hydrothermal (or solvothermal) synthesis relies on dissolving inorganic substances in water or another solvent at temperatures above 100 °C and pressures around 1 atm, followed by crystallization. The key difference is that hydrothermal methods use water, while solvothermal methods employ other solvents. REVO_4_ can be readily prepared this way, though high-temperature calcination is required to enhance luminescence. Reaction parameters such as pressure, temperature, pH, and precursor ratios determine the morphology and size of the final product. The hydrothermal synthesis method provides significant advantages for producing vanadates through multiphase or liquid-phase chemical processes. However, it has notable drawbacks: need for costly autoclaves, safety risks during reactions, and difficulty of monitoring processes directly. For example, GdVO_4_:Ln^3+^ (Ln = Eu, Sm, Dy) microcrystals and nanocrystals with different morphologies, crystal orientations and defects have been synthesized via a simple hydrothermal method in a wide range of pH values into a Teflon-lined stainless autoclave at a temperature of 180 °C for 24 h or 10 h at high pressure [30,31,32].

### 2.4. Sol–Gel Route

Sol–gel synthesis is a versatile wet-chemical method that can be performed through routes such as alkoxide hydrolysis, inorganic gelation, or polymerizable complex processes. It enables molecular-level mixing of precursors, offering advantages over solid-state reactions, including uniform dopant distribution, lower synthesis temperatures, reduced contamination, and control over porosity. Drawbacks include difficulty in removing organic groups and risk of cracks in the final material. Chelating agents play a crucial role, as their type and combustion properties influence the physico-chemical characteristics of the products [33]. The sol–gel method is highly valued due to its relatively low initial cost for producing high-quality materials, its ability to design and control the chemical structure of substances, and its capacity to achieve uniform composition with a large surface area. Despite these advantages, the technique has limitations, including extended reaction times and considerable shrinkage during the dehydration stage [34].

In practice, GdVO_4_ is often synthesized from Gd(OAc)_3_ and NH_4_VO_3_ dissolved in water, mixed in the proper molar ratio with carboxylic acids (e.g., citric, malic, or tartaric acid), then heated with stirring to form a gel. The gel is dried to yield a carboxylate precursor, which is subsequently calcined. In some cases, synthesis can proceed without carboxylic acids. Using different acids allows production of vanadate with different sizes and morphologies. For example, tartaric acid can serve both as a chelating agent and combustion fuel, with calcination temperature and precursor ratios directly affecting particle size and crystallinity [35].

### 2.5. Microwave-Assisted Method

Microwave synthesis employs electromagnetic radiation (0.3–300 GHz) to rapidly decompose precursors and trigger fast nucleation, producing small nanoparticles in a much shorter time. A key requirement is that at least one precursor must absorb microwaves. This method is often combined with hydrothermal synthesis. Microwave irradiation plays a vital role in accelerating reaction kinetics by enabling rapid initial heating, which significantly increases overall reaction rates. This technique produces cleaner products, ensures faster consumption of starting materials, and improves yields. Another advantage lies in the uniform heating and precise control of process parameters, which enhance reproducibility and reliability of reaction conditions. In the field of vanadate synthesis, microwave heating represents a novel and uniform approach that deserves further development. Using green reaction media not only shortens reaction times but also minimizes chemical waste, making it an attractive and sustainable method [36]. Typically, Gd(NO_3_)_3_·6H_2_O and NH_4_VO_3_ solutions are mixed with EDTA at high pH, stirred, and placed in a microwave reactor at 150 °C for 180 min. The resulting powders are collected by centrifugation, washed with water and ethanol, and dried [37,38].

## 3. Structural and Morphological Properties of REVO_4_-Based Materials

### 3.1. Crystal Structure of REVO_4_-Based Materials

**Figure 1:**
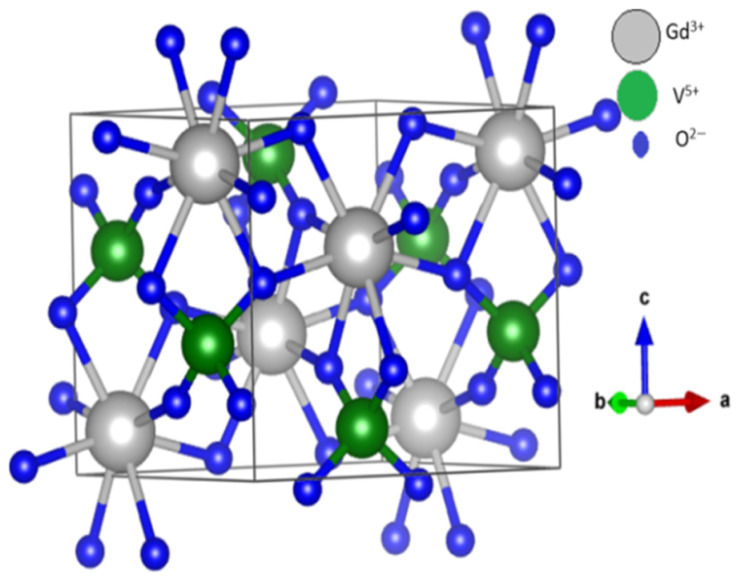
Tetragonal crystal structure of GdVO_4_. Reproduced from Ref. [39] under Institute of Physics and IOP Publishing license.

Materials of the type AVO_4_ (where A = Sc, Y, Bi, or any of the lanthanides La–Lu) adopt a tetragonal zircon-type crystal structure, similar to ZrSiO_4_. In this arrangement, the V^5+^ ions within the vanadate VO_4_^3−^ groups are tetrahedrally coordinated by O^2−^ ions, while the trivalent A^3+^ cations are surrounded by eight O^2−^ ions. The three-dimensional framework is built from alternating AO_8_ distorted dodecahedra in which the A^3+^ cations occupy a non-centrosymmetric crystallographic site with D_2d_ symmetry that shares edges with tetrahedral VO_4_. As an example, the tetragonal crystal structure of GdVO_4_ is presented in Figure 1. This connectivity results in chains of vanadium ions aligned parallel to the c-axis. Bond distances vary within the structure: there are four shorter (2.33 Å) and four longer (2.45 Å) A–O bonds, whereas V–O bonds are all 1.74 Å long, consistent with tetrahedral coordination of V^5+^ ions. Each O^2−^ ion is bonded to two equivalent A^3+^ cations and one V^5+^ ion. Importantly, substitution of different A^3+^ ions does not alter the crystal type, as all AVO_4_ compounds are isostructural. For example, YVO_4_ compound parameters are a = 7.13 Å, b = 7.13 Å, c = 6.30 Å, α = β = γ = 90° and unit cell volume V = 320.53 Å^3^ [39]. Many REVO_4_ compounds exhibit a low-temperature Jahn–Teller distortion, in which their tetragonal crystal structure transforms into an orthorhombic geometry. Interestingly, this type of crystallographic transition has not been observed in YVO_4_ or in GdVO_4_ [40].

Also, the lattice distortion can be induced by the incorporation of a dopant (for example, Eu^3+^ ions) into the host crystal structure. As an example, when Ho^3+^ and Yb^3+^ ions are incorporated into the GdVO_4_ crystal, the structure changes. As the amount of Yb^3+^ increases, one of the diffraction peaks can shift to higher angles because the smaller Ho^3+^ and Yb^3+^ ions replace the larger Gd^3+^ ions, inducing a shrinking of the crystal lattice. A similar effect is recognized when Li^+^ ions are added to the system. Since the ionic radius of Li^+^ is smaller than that of Gd^3+^, it can move easily through the crystal and take the place of Gd^3+^ ions or occupy interstitial sites. The structural parameters of synthesized Ho^3+^/Yb^3+^-doped GdVO_4_ powders with and without Li^+^ co-doping, calculated using Rietveld refinement, were a = 7.175 Å, b = 7.13 Å, c = 6.324 Å, and V = 325.57 Å^3^ for GdVO_4_ with 1%Ho^3+^ and 20%Yb^3+^ and a = 7.166 Å, b = 7.166 Å, c = 6.319 Å, and V = 324.52 Å^3^ for GdVO_4_ with 1%Ho^3+^, 20%Yb^3+^, and 15%Li^+^ [28].

**Figure 2:**
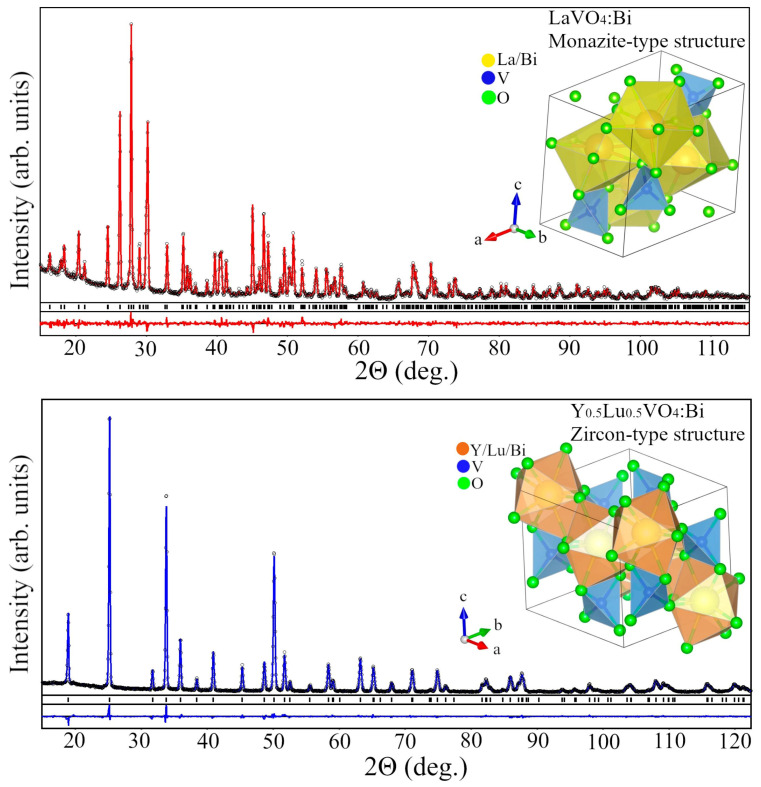
Graphical results of Rietveld refinement of Bi-doped LaVO_4_ (**top**) and Y_0.5_Lu_0.5_VO_4_ (**bottom**) structures. Experimental XRD patterns (black circles) are shown in comparison with the calculated profiles (red and blue lines, respectively). Short vertical bars on the top and bottom panels indicate positions of Bragg’s maxima in the monoclinic *P*2_1_/*n* and tetragonal *I*4_1_/*amd* structures, respectively. Insets show polyhedral views of monazite-type LaVO_4_:Bi and zircon-type Y_0.5_Lu_0.5_VO_4_:Bi structures. Reproduced from Ref. [43] under Creative Commons CC BY license.

Figure 2 depicts the difference in the structural configuration of the monoclinic LaVO_4_ and Y_0.5_La_0.5_VO_4_ structures doped with Bi^3+^ together with Rietveld refinement. In the LaVO_4_ structure, La atoms are coordinated in a hexahedral arrangement with six oxygen atoms, while V atoms are coordinated to four oxygen atoms, forming a tetrahedral geometry. This host lattice contains two types of cations, La^3+^ with ionic radius r = 1.16 Å and V^5+^ with ionic radius r = 0.35 Å, for coordination number 8. The dopant ion Bi^3+^ (with ionic radius r = 1.17 Å) for coordination number 8 shares the same valence state as La^3+^ and exhibits similar ionic sizes and coordination environments and preferentially substitutes at La^3+^ sites within the lattice. The Y_0.5_La_0.5_VO_4_ structure crystalizes in a tetragonal zircon-type crystal structure, as mentioned and explained above for GdVO_4_ [41,42,43].

### 3.2. Morphology of Undoped and Ln^3+^-Doped REVO_4_ Phosphors

The development of modern optoelectronic devices strongly depends on the advancement of luminescent materials with improved characteristics. These materials must exhibit specific functional properties such as brightness, resolution, spectral energy distribution and lifetime under practical operating conditions. Such properties are determined by the structural and morphological features of the material used. Depending on the intended application, synthesized luminescent Ln^3+^-doped REVO_4_ phosphors should possess precisely defined functional characteristics. Among them, uniform particle size distribution and spherical morphology without agglomeration are particularly critical [44,45].

Precise control over nanoparticle size, uniformity, distribution and surface area enables significant enhancement of optical properties, such as lifetimes of intermediate energy levels, dopant ion emission at specific wavelengths, and reduction in light scattering when particle dimensions are smaller than the wavelength of incident light. Luminescent materials at the nanoscale are especially important because they bridge the gap between molecular and micron scales, offering unique opportunities for advanced applications. Their effectiveness arises from a high surface-to-volume ratio, which ensures that a large fraction of atoms remain available for interaction with surrounding molecules [46].

Furthermore, the small particle size provides greater flexibility for manipulation and facilitates efficient doping with activator ions. Since many dopant ions are located near particle surfaces in asymmetric crystalline environments, their emission behavior differs from that of ions in regular crystallographic positions. This distinction opens pathways to novel and tunable optical effects, positioning nanostructured luminescent materials as a promising frontier in optoelectronics [47].

As mentioned above, the morphology of Ln^3+^-doped REVO_4_ is strongly influenced by the preparation method and reaction parameters, such as reaction temperature, pressure, concentration of precursor solutions, pH value, kind of solvent, aging time, the type, concentration and distribution of Ln^3+^ dopant ions, and addition of capping agent or surfactant. For example, GdVO_4_:Eu^3+^/Sm^3+^ and GdVO_4_:Ho^3+^/Yb^3+^ obtained with solid-state reaction synthesis show that the powders contain chunks with irregular spheres with an average diameter ranging from 1 µm to 8 µm [28,48].

Using the sol–gel route, the GdVO_4_ nanomaterial can be synthesized using different carboxylic acids, resulting in a homogeneous distribution of spherical particles with average sizes ranging from 50 to 100 nm [49]. In another approach, a tartaric acid-assisted sol–gel method was employed, where tartaric acid acted simultaneously as a chelating agent and as an additional fuel during precursor combustion. Key synthesis parameters, such as calcination temperature and the molar ratio between total metal ions and tartaric acid, directly influence the particle size and crystallinity of the final product, consisting of spherical nanoparticles with diameters between 20 and 30 nm [35].

GdVO_4_ nano- and microcrystals with diverse morphologies and sizes were successfully synthesized using a hydrothermal process and trisodium citrate (Na_3_Cit) as the chelating ligand. By employing Gd(NO_3_)_3_ and Na_3_VO_4_ as precursors together with Na_3_Cit in varying molar ratios relative to Gd^3+^ ions, different morphologies and particle sizes were obtained. For instance, when the molar ratio of Na_3_Cit to Gd^3+^ was 4:1, uniform pancake-like microstructures were produced, with an average thickness of 200 nm and a diameter of approximately of 1 µm [31]. In another approach, hydrothermal synthesis under varying pH conditions, reaction media and reaction times yielded Eu^3+^-doped GdVO_4_ phosphors with a wide range of morphologies, such as rhombic, spherical, and cubic structures, as well as irregular short nanorods [30]. Applying various reaction components, such as EDTA-Na_2_ and EDTA, and adjusting the pH of the solution, the hydrothermal method allows one to tune the shape of GdVO_4_ into short and long nanowires, nanorods, nanoparticles and spheres [50].

Both as-prepared GdVO_4_:Ho^3+^/Yb^3+^ samples and those re-heated at 300 °C, synthesized by the coprecipitation method, reveal bundles composed of 5–6 individual nanorods, each approximately 5 nm in diameter and up to 20 nm in length, aligned in different orientations. When the same samples were annealed at 600 °C, the nanorods transformed into single ellipsoidal particles with an average size of about 20 nm, while at higher annealing temperatures of 800 °C and 1000 °C, the morphology changed further, yielding irregular spherical particles approximately 100 nm in size along with elongated rod-like structures [22].

The YVO_4_ nanoparticles synthesized using a simple microwave irradiation process exhibited a size in the range of 5–18 nm, which was extremely dependent on the pH value of the solution [51]. On the other hand, the YVO_4_:Bi^3+^/Eu^3+^ materials synthesized by a microwave and ultrasonic radiation-activated technique had a pronounced spherical shape with a predominant diameter in the range of 20–40 nm [52].

**Figure 3:**
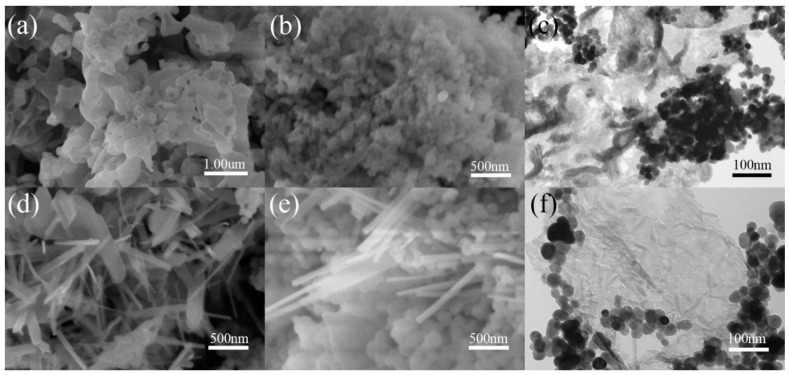
SEM images of (**a**) g-C_3_N_4_, (**b**) GdVO_4_, (**d**) LaVO_4_, and (**e**) LaVO_4_/g-C_3_N_4_ and TEM images of (**c**) GdVO_4_/g-C_3_N_4_ and (**f**) LaVO_4_/g-C_3_N_4_. Reproduced from Ref. [53] under Creative Commons CC BY license.

As a further example, SEM and TEM images of MVO_4_/g-C_3_N_4_ (M = La, Gd) prepared by the hydrothermal method are given in Figure 3. Pure g-C_3_N_4_ exhibits a nanosheet structure with a smooth surface (Figure 3a), while pure GdVO_4_ powders display a coral-like morphology (Figure 3b). The TEM image of the GdVO_4_/g-C_3_N_4_ composite (Figure 3c) reveals two distinct components: the black coral-like structures corresponding to GdVO_4_, and the French-gray sheet-like structures belonging to g-C_3_N_4_. On the other hand, pure LaVO_4_ demonstrates a pin-like nanostructure (Figure 3d). Figure 3e,f illustrate the LaVO_4_/g-C_3_N_4_ composite, where both LaVO_4_ and g-C_3_N_4_ are clearly distinguishable, with LaVO_4_ particles well adhered to the g-C_3_N_4_ surface. Thus, the constitution of MVO_4_/g-C_3_N_4_ (M = La, Gd) composites is readily identifiable, which facilitates efficient charge carrier transport compared to pure MVO_4_ (M = La, Gd) [53].

## 4. Optical Properties of Undoped and Ln^3+^-Doped REVO_4_ Materials

The lanthanides, spanning lanthanum (Z = 57) to lutetium (Z = 71), have a general electronic configuration of [Xe]6s^2^5d^1^4f^n^. Typically, the 5d electron shifts into the 4f shell, giving 4f^n+1^, though exceptions exist (e.g., Gd). As atomic number increases, the 4f orbitals contract inward, a phenomenon called the lanthanide contraction, making them behave like inner electrons and limiting their role in bonding. This results in similar chemical properties across the series, dominated by outer valence electrons. Upon ionization, lanthanides form stable +3 ions ([Xe]4f^n^), with 5s^2^5p^6^ orbitals shielding the 4f electrons, so crystal field effects remain weak and are treated with perturbation theory [54]. Inorganic materials doped with Ln^3+^ are essential across numerous fields, owing to their wide-ranging applications: LED diodes, luminescent monitors/electromagnetic displays, lasers, thin-film phosphors, drug delivery systems, luminescence thermometry, chemical sensing, biosensing, infrared fluorescence bioimaging, photothermal therapy, gas sensing, anti-counterfeiting technologies and so on.

REVO_4_ (RE = Y, Gd, Lu and La) compounds are widely recognized as excellent host materials for various dopant ions, owing to their ability to be efficiently excited by UV radiation and their favorable charge-transfer energy. Because charge transfer from VO_4_^3−^ groups to optically active Ln^3+^ ions is highly efficient, luminescence can be achieved even at extremely low dopant concentrations [55].

Ln^3+^-doped REVO_4_ luminescent materials can be classified in several ways depending on their properties and targeted applications. From a functional perspective, these materials fall into three main categories: lighting materials, display materials, and detection/sensing materials. When they are classified by excitation source, they include photoluminescent, X-ray-excited luminescent, electroluminescent, and high-energy photon-excited materials. Based on the photon emission dynamics, these materials are distinguished as either downconverters or upconverters [47,48,56].

### 4.1. Downconversion of Ln^3+^-Doped REVO_4_ Phosphors and Sensing Applications

Stokes luminescence, also known as downconversion (DC), is a process in which matter absorbs photons of higher energy and re-emits photons of lower energy. DC luminescence follows Stokes’ law, describing the conversion of high-energy excitation into lower-energy emission [57,58].

**Figure 4:**
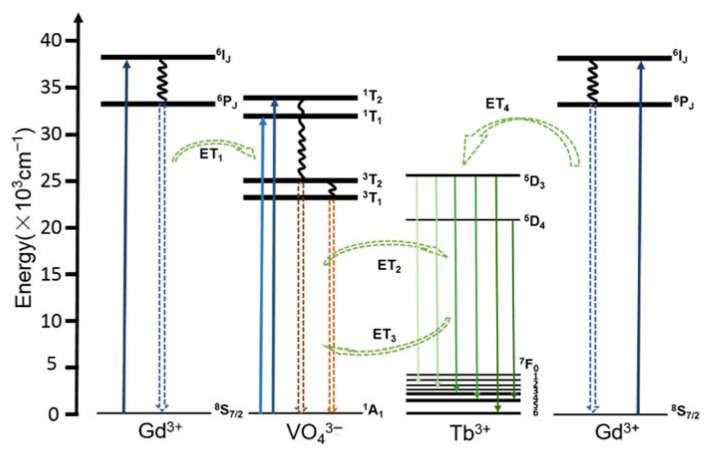
Schematic diagram of the energy transfer process in GdVO_4_:Tb samples. Reproduced from Ref. [59] under Creative Commons CC BY license.

As an example for REVO_4_-based DC-luminescent materials, the schematic diagram of the energy transfer process in GdVO_4_:Tb samples is given in Figure 4. The VO_4_^3−^→Tb^3^ one-electron charge transfer takes place between the 2p orbital of oxygen (O^2−^) and the vacant 3d orbital of the central vanadium (V^5+^) in the tetrahedral VO_4_^3−^ with Td symmetry. According to the molecular orbital theory, energy levels involved are the ground ^1^A_1_ state and the excited ^1^T_1_, ^1^T_2_, ^3^T_1_ and ^3^T_2_ states. The transitions from ^1^A_1_ level to the ^1^T_1_ and ^1^T_2_ generate a broad and intense charge-transfer absorption band in the UV region [11]. At the same time, weak absorption features indicate a low-efficiency reverse transfer process from Tb^3+^ to VO_4_^3−^. There are four possible energy transfer (ET) pathways, ET_1_ (Gd^3+^→VO_4_^3−^), ET_2_ (VO_4_^3−^→Tb^3+^), ET_3_ (Tb^3+^→VO_4_^3−^, weak) and ET_4_ (Gd^3+^→VO_4_^3−^→Tb^3+^), which demonstrate cooperative interactions among Gd^3+^, VO_4_^3−^ and Tb^3+^ ions. Overall, VO_4_^3−^→Tb^3+^ transfer was identified as the dominant process, supported by spectral shifts, broadened emission bands, and lifetime analysis [59].

**Figure 5:**
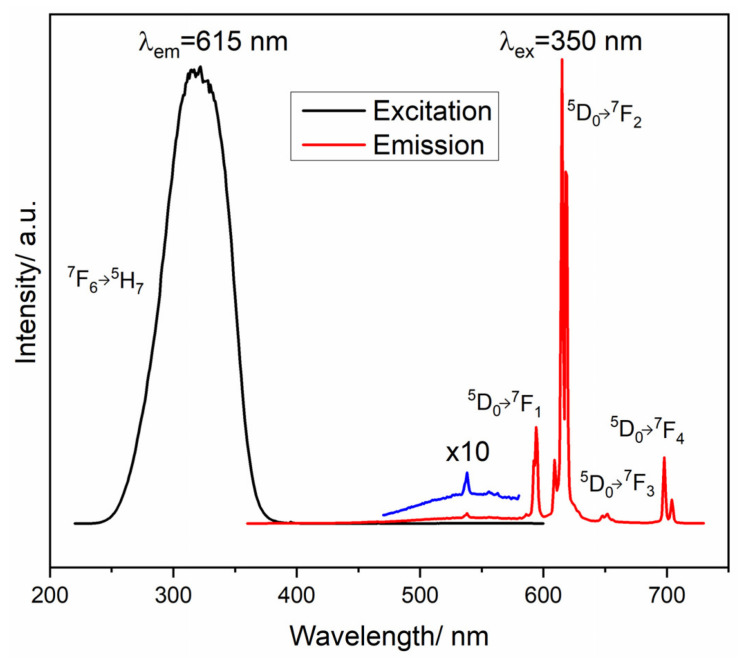
Excitation and emission spectra of LuVO_4_ films with 2.5 at. % Eu^3+^ and 1.5 at. % Bi^3+^ after 3 h of annealing at 1000 °C. Reproduced from Ref. [61] under Creative Commons CC BY license.

Eu^3+^ and Bi^3+^ ion co-doped LuVO_4_ thin films annealed at 1000 °C exhibit pronounced luminescent behavior. The Eu^3+^ ions generate intense red emission via the ^5^D_0_→^7^F_2_ transition with a similar feature to the GdVO_4_:Eu^3+^ samples obtained by coprecipitation synthesis and additionally annealed at 1000 °C [60]. As an example, excitation and emission spectra of LuVO_4_:Eu^3+^/Bi^3+^ films are given in Figure 5.

Under 350 nm excitation, emission peaks at 594, 615, 650, and 700 nm are produced, corresponding to Eu^3+^ transitions ^5^D_0_→^7^F_J_ (J = 1, 2, 3, 4), while the most prominent emission is observed at 615 nm. The Bi^3+^ ions act as sensitizers by absorbing UV radiation and transferring the energy to Eu^3+^ ions. The characteristic Bi^3+^ emission band at 550 nm is significantly reduced in co-doped samples, confirming efficient Bi^3+^→Eu^3+^ energy transfer. LuVO_4_ thin films exhibit stable and tunable luminescence, making them highly promising for optoelectronic applications such as displays and light emitters, as well as efficient phosphors for LEDs, particularly in warm-white lighting. Their properties also open opportunities in optical sensing and biological imaging, where reliable luminescence performance is essential [61]. Analysis of the intensity ratio R = I(^5^D_0_→^7^F_2_)/I(^5^D_0_→^7^F_1_) demonstrates that Bi^3+^ promotes Eu^3+^ occupation at lower-symmetry sites. According to Dexter’s theory, quadrupole–quadrupole interactions dominate the transfer mechanism, consistent with other Bi^3+^→Eu^3+^ systems [62].

The Eu^3+^ ions are frequently employed as luminescent activators for bioapplications (biodetection, bioimaging and biosensing) and chemical sensing, due to their unique luminescence properties. Due to the different crystal field surroundings, the Eu^3+^ ions situated at multiple sites in different materials exhibit distinct photoluminescent spectra and photoluminescent decays. The long-lived luminescence of Eu^3+^ ions is highly advantageous for background-free, time-resolved photoluminescence biodetection. Eu^3+^-activated nanomaterials have demonstrated broad applicability in both heterogeneous and homogeneous biodetection, in vitro and in vivo bioimaging, and anti-aging, antibacterial, anticancer and antioxidant effects [63,64]. GdVO_4_:Eu^3+^ nanoparticles are excellent candidates for multifunctional material development, as they can perform diverse and polyvalent functions.

**Figure 6:**
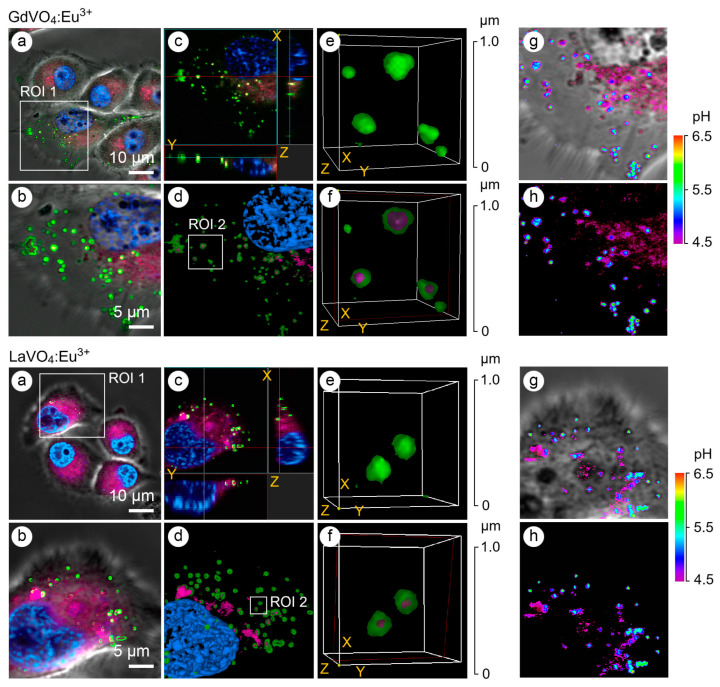
Representative Laser Scanning Confocal Microscopy (LSCM) images of the endosome-positive L929 cells following treatment with GdVO_4_Eu^3+^ and LaVO_4_Eu^3+^ nanoparticles at 20 mg/L for 1 h. The L929 cells were transfected with GFP-Rab5a/GFP-Rab7a and, 24 h after transfection, the cells were exposed to the investigated NPs. The Rab5a and Rab7a proteins were markers of the early and late endosomes, respectively. The GFP-Rab5a/GFP-Rab7a-positive endosomes (green) and NPs’ autofluorescence signals (magenta) were detected. The cell nuclei are shown in blue (DAPI staining). Single-optic section phase contrast and fluorescence-merged imaging (Panel (**a**)); magnified single-optic section fluorescence imaging of the ROI1 (Panel (**b**)); orthogonal XZ and YZ projections of the ROI1 (Panel (**c**)); 3D reconstruction of the ROI1 (Panel (**d**)); complete 3D reconstruction of the endosomes without and with the NPs’ fluorescence channel (magenta) of the ROI2 (Panel (**e**)); cross section of the endosomes (red frame) demonstrating the intra-endosomal localization of the NPs (magenta) of the ROI2 (Panel (**f**)); and pseudocolored and phase-contrast imaging of the endosomal pH using the LysoSensor ratiometric probe (molecular probes, L22460) of the ROI1 (Panels (**g**,**h**)). Ratiometric pseudocolored images were constructed from two emission images at 450 ± 33 nm and 510 ± 20 nm, respectively. Both were excited at 365 ± 8 nm. The cells were preliminarily exposed to pH calibration buffers (pH 4.5–6.5). Reproduced from Ref. [65] under Creative Commons CC BY license.

Redox-active GdVO_4_:Eu^3+^ and LaVO_4_:Eu^3+^ nanoparticles can intensify damage in oxidatively stressed L929 cells, even at concentrations that remain non-toxic to normal cells. This effect is linked to the internalization of the nanoparticles and is mediated through activation of the intrinsic mitochondrial apoptotic pathway, excessive reactive oxygen species (ROS) generation and Ca^2+^ signaling. These results highlight the potential of GdVO_4_:Eu^3+^ and LaVO_4_:Eu^3+^ nanoparticles as promising candidates for use as anticancer agents, as depicted in Figure 6 [65].

A ^64^Cu-labeled multifunctional nanoprobe targeting integrin α_2_β_1_ (often used as a metastasis suppressor) was developed using GdVO_4_:Eu^3+^ two-dimensional tetragonal nanosheets for in vitro fluorescence studies, in vivo magnetic resonance imaging (MRI) and micro-positron emission tomography (PET) imaging of prostate cancer. The unique water solubility and biocompatibility make GdVO_4_:Eu^3+^ highly versatile for biomedical applications [66], with strong potential as a radionuclide carrier and contrast agent for theranostic applications [67,68].

Due to their negative surface potential, GdVO_4_:Eu^3+^ nanoparticles strongly adsorb metal cations. Among common blood ions, Cu^2+^ uniquely induces distinct fluorescence quenching. GdVO_4_:Eu^3+^ nanoparticles are promising magnetic/fluorescent multimodal probes for Cu^2+^ ion detection in blood [69]. Colloidal GdVO_4_:Eu^3+^@SiO_2_ nanocrystals could find a promising application for highly selective and sensitive detection of Cu^2+^ ions in an environmental or biological sample [70]. Also, Eu^3+^activated ultra-small nanoparticles (EuVO_4_ and GdVO_4_:Eu^3+^) have the potential to be used for the detection of pesticides in environmental and biomedical fields, due to photoluminescence quenching [71]. Hydrogen peroxide (H_2_O_2_) acts as a strong quencher of ultra-small (2–3 nm) GdVO_4_:Eu^3+^ fluorescence. The observed Eu^3+^ luminescence quenching is found to be more effective with increasing concentrations of H_2_O_2_. To evaluate selectivity for H_2_O_2_, several potentially interfering ions were also tested. Common physiological ions such as Ca^2+^, Zn^2+^ and Mg^2+^ did not affect fluorescence intensity, while Cu^2+^ and Fe^3+^ quenched even more efficiently than H_2_O_2_ [72]. Two primary mechanisms were identified as responsible for the quenching of Eu^3+^ luminescence in GdVO_4_:Eu^3+^ nanoparticles: (i) reduction in the efficiency of non-radiative resonance energy transfer from the vanadate groups to Eu^3+^ ions, caused by scattering effects introduced by V^4+^ ions; (ii) direct luminescence quenching of Eu^3+^ ions by –OH groups formed on the nanoparticle surface as a result of H_2_O_2_ decomposition [73].

The additional sensing application and distribution of Gd_0.6_Eu_0.4_VO_4_ nanoparticles was investigated with XRF spectroscopy after injection of their colloidal solutions into mouse ear pinnae. The distribution of the nanoparticles was mapped by raster scanning the sample and detecting the V K_α_, Gd L_α_, and Eu L_α_ fluorescence emissions. It was found that all three elements coexist and therefore no short-term out-diffusion of those elements from the nanoparticles to the tissues takes place. These results provided a proof-of-concept that XRF mapping and spectroscopy are excellent tools for the determination of the long-term fate of Gd_0.6_Eu_0.4_VO_4_ nanoparticles in tissue in terms of element leaching and stability for diagnostic and therapeutic purposes [74].

### 4.2. Upconversion of Ln^3+^-Doped REVO_4_ Phosphors and Sensing Applications

Upconversion (UC) luminescence is a non-linear anti-Stokes process with low-energy excitation light (NIR region). In recent years, UC materials excited by NIR light illumination and emitting in the visible (Vis) region have received much attention. Typically, UC materials are composed of a host material with low phonon energy, such as YVO_4_, GdVO_4_, LuVO_4_ and LaVO_4_ and sensitizer ions (commonly Yb^3+^), along with activator ions (most often Er^3+^, Tm^3+^, or Ho^3+^, or their combination). Yb^3+^-sensitized UC materials are typically excited at 980 nm. The Yb^3+^ ion possesses a simple energy structure with only two states, the ground state (^2^F_7/2_) and the excited state (^2^F_5/2_), separated by an energy gap of approximately 10,000 cm^−1^. Yb^3+^ acts as the sensitizer due to its strong absorption cross-section at 980 nm and efficient energy transfer to Er^3+^, while Er^3+^ provides the emission. Importantly, Yb^3+^ absorbs in the NIR region where inexpensive laser diodes operate efficiently. This absorption enhances luminescence efficiency by transferring excitation energy to Er^3+^, Ho^3+^, or Tm^3+^ ions [75].

The intensity of upconversion emission is strongly influenced by several factors, including temperature, particle morphology, surface characteristics, and concentration ratio between dopant ions (Er^3+^, Ho^3+^, Tm^3+^) and sensitizer ions (Yb^3+^). In the GdVO_4_ host matrix, varying the concentration ratio of Yb^3+^ to Er^3+^ alters the balance between green and red emission. It is important to note that literature indicates only a few Er^3+^ or Yb^3+^/Er^3+^-doped inorganic UC materials which emit intense green emission under NIR excitation. Specifically, increasing the Er^3+^ concentration enhances the green emission in doped GdVO_4_ and YVO_4_ materials. This effect can be attributed to perturbations in site symmetry, which promote stronger emission from the ^2^H_11/2_ state. The perturbation arises from the hypersensitive nature of the ^2^H_11/2_ state and the difference in ionic radii between Yb^3+^ and Gd^3+^ ions, leading to modified local environments that favor enhanced luminescence [76,77]. The UC mechanism involves Yb^3+^ absorbing most of the excitation energy and transferring it to Er^3+^, whose long-lived excited states enable multiphoton absorption and radiative relaxation, producing green and red emissions. Nanoparticles show weaker UC intensity compared to bulk material, though the emission band shapes and green-to-red ratios remain unchanged [78].

**Figure 7:**
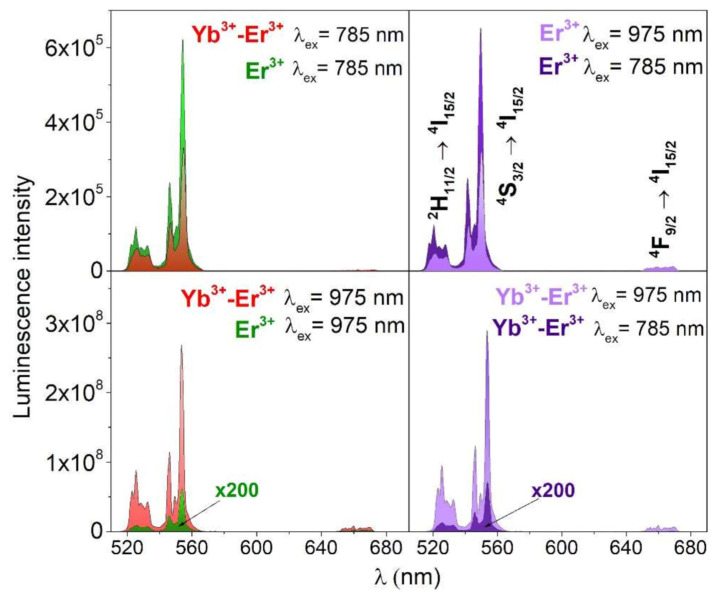
Non-normalized UC emission spectra of the obtained nanomaterials YVO_4_:Er^3+^ and YVO_4_:Yb^3+^,Er^3+^ under λ_ex_ = 785 or 975 nm (≈50 W/cm^2^). Reproduced from Ref. [79] under Creative Commons Attribution (CC BY) license.

Figure 7 demonstrates how excitation wavelength and elemental composition influence the green UC emission intensity of YVO_4_:Er^3+^ and YVO_4_:Er^3+^,Yb^3+^ nanomaterials. The excitation of the samples at 785 nm resulted in higher UC emission intensity for the single-doped YVO_4_:Er^3+^ compound. In contrast, the most intense green UC luminescence is observed in YVO_4_:Er^3+^,Yb^3+^ under 975 nm excitation due to the large absorption cross-section of Yb^3+^ ions (^2^F_7/2_ → ^2^F_5/2_ transition) and the effective energy transfer UC from the sensitizing Yb^3+^ ions to the emitting Er^3+^ ions [79]. Also, the laser pump power can affect the intensity of the Er^3+^ emission measured for large nanoparticle ensembles and single particle in the YVO_4_:Yb,Er system [80].

The incorporation of Ho^3+^ ions into the matrix, together with Yb^3+^ ions, is particularly effective due to the favorable energy level distribution of Ho^3+^ ions. Radiative transitions from the ^5^S_2_/^5^F_4_ level of Ho^3+^ to the ground state (^5^I_8_) produce green emission around 545 nm, while transitions from the ^5^F_5_ level to ^5^I_8_ yield red emission near 650 nm under UV and NIR excitation, respectively. The intensity of red UC emission originating from the ^5^F_5_ → ^5^I_8_ transition of Ho^3+^ increases consistently with higher Yb^3+^ concentrations. Typically, strong red UC emission in Ho^3+^/Yb^3+^ systems requires relatively high Ho^3+^ concentrations, explained by cross-relaxation processes between Ho^3+^ energy levels that suppress green emission [81,82].

**Figure 8:**
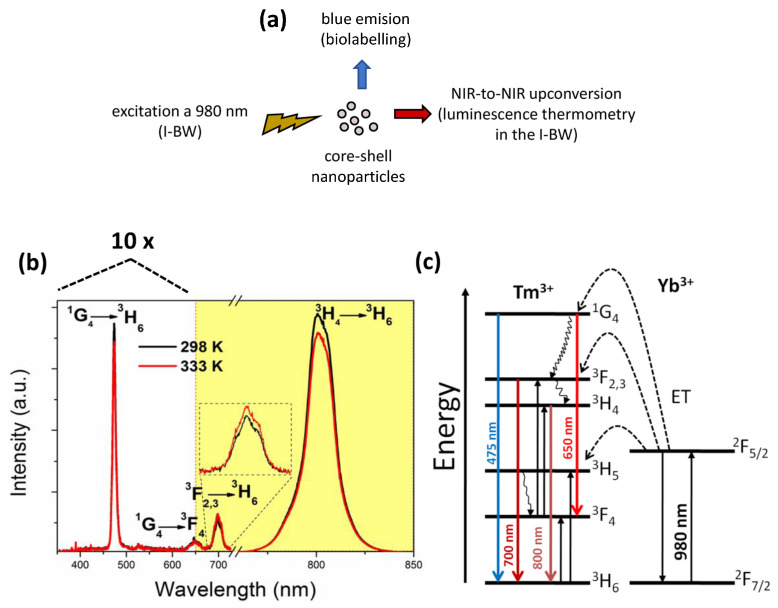
(**a**) Schematic representation of the pumping and emission bands generated by the Tm,Yb:GdVO_4_@SiO_2_ core–shell nanoparticles. (**b**) Upconversion emission spectra of the Tm,Yb:GdVO_4_@SiO_2_ core–shell nanoparticles at room temperature and at 333 K. The inset shows the magnification of the peak located at 700 nm. (**c**) Energy level diagram of Tm^3+^ and Yb^3+^ ions in GdVO_4_, indicating the absorption, energy transfer and emission pathways. Reproduced from Ref. [84] under Creative Commons Attribution (CC BY) license.

GdVO_4_:Tm^3+^/Yb^3+^ material, under 980 nm excitation, exhibits UC luminescence in three distinct regions: a dominant blue emission at 475 nm arising from the ^1^G_4_ → ^3^H_6_ transition of Tm^3+^ions, a weaker red emission at 650 nm attributed to the ^1^G_4_ → ^3^F_4_ transition and an infrared emission at 808 nm corresponding to the ^3^H_4_ → ^3^H_6_ transition of Tm^3+^ ions [83]. Figure 8 summarizes the structure and luminescence behavior of Tm^3+^,Yb^3+^:GdVO_4_@SiO_2_ core–shell nanoparticles, which can serve as biolabels in the visible range and luminescence thermometers within the first biological window (Figure 8a). Under 980 nm excitation, the UC emission spectra reveal that most bands decrease in intensity with rising temperature, except the 700 nm band, which slightly increases (Figure 8b). The energy transfer mechanism (Figure 8c) involves sequential absorption by Yb^3+^ ions (^2^F_7/2_ → ^2^F_5/2_) and transfer to Tm^3+^ levels, ultimately populating ^1^G_4_ and producing blue (475 nm) and red (650 nm) emissions, while transitions from ^3^F_3_ and ^3^H_4_ yield emissions at 700 nm and 800 nm. Non-radiative relaxation, Tm–Tm cross-relaxation and efficient population of the ^3^H_4_ level can explain the strong NIR emission observed at 800 nm and the temperature-dependent spectral behavior [84].

It is common to use a 980 nm excitation source for UC emission, but this wavelength has drawbacks in biomedical applications, including strong water absorption, limited tissue penetration and local heating that can damage cells. To overcome these issues, research has shifted toward shorter excitation wavelengths, particularly 808 nm, which lies within the biological NIR windows (650–950 and 1100–1750 nm) where tissues are more transparent. The second NIR window is especially attractive for fluorescence imaging due to deeper penetration, higher resolution, and reduced scattering. At 808 nm, water absorption is lower, penetration is good in superficial to moderately deep tissues, and heating effects are minimized [85,86]. The excitation wavelength shift is possible by substituting Yb^3+^ ions with Nd^3+^ ions, which have a strong absorption band centered at 808 nm. UC emission spectra of GdVO_4_:Nd^3+^/Er^3+^ and GdVO_4_:Nd^3+^/Ho^3+^ systems under 808 nm excitation show three bands: green emission (520–565 nm, max ~540 nm), red emission (570–630 nm, max ~597 nm), and another red band (640–680 nm, max ~675 nm). The dominant red emission at 597 nm corresponds to the Nd^3+^ (^4^G_7/2_ → ^4^I_11_/_2_) transition, while the other bands arise from Er^3+^ and Ho^3+^ transitions [58]. GdVO_4_ single crystals doped with Nd^3+^ ions under 808 nm continuous-wave laser excitation across varying temperatures have been studied for optical thermometry. The excitation pathway involves ground-state absorption, non-radiative relaxation, excited-state absorption and energy-transfer upconversion, which enhances higher-level populations. As temperature increases, phonon-assisted processes such as cross relaxation and non-radiative transitions become more efficient, leading to dramatic emission intensity enhancements at 400 °C compared to room temperature [87]. YVO_4_:Nd^3+^ nanophosphors can also be used as NIR-to-NIR thermal sensors in a wide temperature range [88]. Ultra-small stoichiometric NdVO_4_ nanoparticles (3–4 nm) dispersed in water exhibit long-term stability, large absorption cross-sections at 808 nm, and excellent biocompatibility, making them promising multifunctional agents for biomedical imaging and in vivo photothermal cancer therapy in a mouse model. A concentrated colloidal dispersion of NdVO_4_ nanoparticles in Tris-buffered saline (TBS) was subcutaneously injected, and infrared fluorescence imaging with an InGaAs CCD camera enabled precise localization of the injection site [89,90].

Due to the unique 4f-electron structure of RE ions, UC nanomaterials have been widely investigated for biomedical and materials science sensing applications. Due to remarkable optical, electronic and magnetic properties, these materials can be used in biomedicine, luminescence imaging, cell labeling probes, magnetic resonance imaging (MRI), photodynamic therapy (PDT), chemotherapy, single-photon emission computed tomography (SPECT), X-ray computed tomography (CT), optical biosensing/biodetection, and many other sensing technologies. In biomedical contexts, UC nanoparticles offer several advantages: deeper penetration into biological tissues, minimal autofluorescence background, reduced scattering and absorption, excellent photostability and negligible photoblinking [91,92].

The unique ability of UC nanoparticles to function as MRI contrast agents, coupled with their strong tissue penetration and prolonged circulation, enables highly sensitive imaging of biological targets. Beyond diagnostics, these nanoparticles can be precisely engineered in size and morphology to act as multifunctional platforms, supporting both imaging and therapeutic applications such as photodynamic therapy, photothermal therapy, and chemotherapy. Their luminescent responsiveness to temperature further broadens their utility, making them valuable tools for thermometry and sensor development. Altogether, UC nanoparticles represent versatile systems at the intersection of medical innovation and optoelectronic technology [93,94].

YVO_4_:Er^3+^/Eu^3+^/Yb^3+^ nanoparticles with an average size of ~100 nm, synthesized using melamine formaldehyde as a template, can serve as dual-mode excitation materials for bioimaging and have been successfully applied in vitro for imaging HeLa cells. Cytotoxicity was evaluated using the methyl thiazolyl tetrazolium (MTT) assay, confirming that functionalized YVO particles exhibit low cytotoxicity, supporting their potential biomedical applications [95]. GdVO_4_@SiO_2_:Tm/Yb core–shell structures demonstrate excellent thermal sensitivity and resolution, making them suitable as intracellular thermal probes for measuring temperature within living HeLa cells [84].

**Figure 9:**
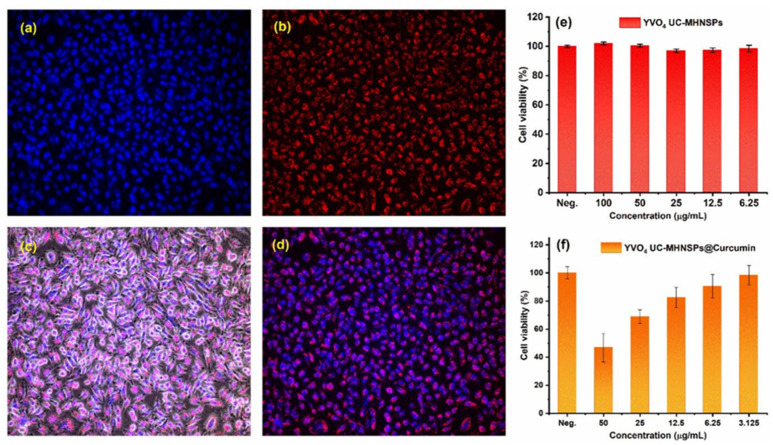
In vitro fluorescence images of YVO_4_:Er^3+^/Yb^3+^ UC-MHNSPs uptake by HeLa cell lines: (**a**) nuclear stain (Hoechst 33342), (**b**) red emission from YVO_4_:Er^3+^/Yb^3+^ UC-MHNSPs, (**c**,**d**) merged fluorescence bright-field and dark-field pictures of HeLa cells under 980 nm excitation. (**e**) Cell viability assessed by CCK-8 assay after incubating with various quantities of YVO_4_:Er^3+^/Yb^3+^ UC-MHNSPs for 24 h and (**f**) antitumor activity of YVO_4_:Er^3+^/Yb^3+^ UC-MHNSPs after conjugation with different quantities of curcumin. Reproduced from Ref. [99] under Creative Commons Attribution (CC BY) license.

YVO_4_:Nd^3+^ nanoparticles have emerged as multifunctional nanotheranostic agents, enabling fluorescence imaging, magnetic resonance imaging, and enhanced sonodynamic therapy of orthotopic gliomas. Similarly, GdVO_4_:Nd^3+^ particles are promising candidates for time-gated bioimaging [96,97,98]. Figure 9 illustrates in vitro imaging studies of YVO_4_:Er^3+^/Yb^3+^ UC-MHNSPs, highlighting their permeability and translocation in HeLa cell lines [99].

**Figure 10:**
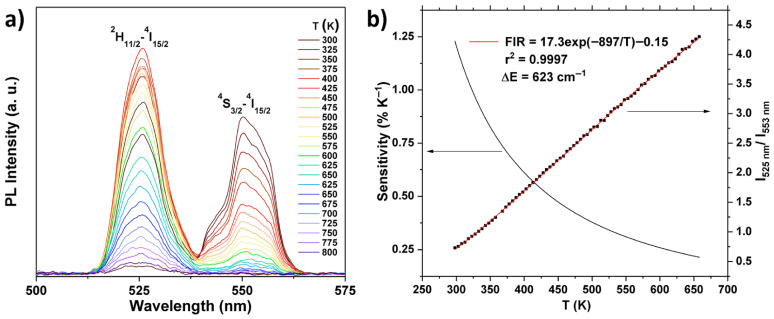
(**a**) Emission spectra of YVO_4_:1%Er^3+^ in the temperature range 300–800 K. (**b**) The temperature dependence of the FIR between the emission peaks at 525 nm (^2^H_11/2_–^4^I_15/2_) and 553 nm (^4^S_3/2_–^4^I_15/2_). The red line shows the best fit for the experimental data (black squares) to the equation: FIR. The black solid line shows the corresponding relative temperature sensitivity. Reproduced from Ref. [102] under Creative Commons Attribution 4.0 International License.

REVO_4_-based UC nanomaterials have been studied as sensors for luminescence thermometry. Temperature monitoring can be achieved by remotely detecting changes in nanomaterials’ luminescent properties, offering valuable their applications in biomedicine, micro/nanoelectronics and integrated photonics [100]. Temperature sensing with UC nanoparticles is of particular interest for biomedicine, because the excitation typically occurs in the NIR spectral region, and autofluorescence from biological material does not affect the measurements. In this context, GdVO_4_:Er^3+^/Yb^3+^ and YVO_4_:Ho^3+^/Yb^3+^ nanoparticles have been investigated for temperature sensing, with their upconversion emission spectra recorded across the range of 307–473 K and 12–300 K, respectively [78,101]. Figure 10a presents the emission spectra of YVO_4_:1%Er^3+^ in the temperature range 300–800 K together with temperature dependence of the fluorescence intensity ratio (FIR) between the emission peaks at 525 nm (^2^H_11/2_–^4^I_15/2_) and 553 nm (^4^S_3/2_–^4^I_15/2_) [102]. Because these two closely separated levels show Boltzmann-type relative population, the integrated FIR of transitions from the ^2^H_11/2_ and ^4^S_3/2_ levels to the ground level ^4^I_15/2_ can be approximated using Boltzmann distribution as follows [103]:

(1) $$FIR=\frac{{{I(}^{2}H}_{11/2}{\to^{4}I}_{15/2\;})}{{{I(}^{4}S}_{3/2}{\to^{4}I}_{15/2\;})}=\frac{g_{H}{\;A}_{H}\;{h\nu }_{H}}{g_{S}{\;A}_{S}\;{h\nu }_{S}}\exp\;(-\;\frac{\Delta E}{kT})=B\exp\;(-\;\frac{\Delta E}{kT})$$

where $g_{H}$ and $g_{S}$ are the degeneracies of the ^2^H_11/2_ and ^4^S_3/2_ levels, respectively; $A_{H}$, $A_{S}$, $\nu_{H}$ and $\nu_{S}$ are the spontaneous emission rates and frequencies of the ^2^H_11/2_ → ^4^I_15/2_ and ^4^S_3/2_ → ^4^I_15/2_ transitions, respectively; h is the Planck constant; k is the Boltzmann constant; and T is the absolute temperature. Equation (1) can be expressed as follows:

(2) $$ln(FIR)=ln(B)+(-\;\frac{\Delta E}{kT})=ln(B)+(-\;\frac{C}{T})$$

where B and C are the constants that need to be determined. Fitting the experimental data with Equation (2) demonstrates a strong correlation between theory and experiment, consistent with previous reports on thermometry based on Er^3+^ UC emission [103,104]. The relative sensor sensitivity, S_r_ [in %K^−1^], of the luminescent probe, is defined as relative change in the FIR with temperature (Figure 10b):

(3) $${\;S}_{r}=\frac{1}{\mathrm{FIR}}\frac{\Delta FIR}{\Delta T}\times 100\%$$

The highest relative sensitivity was achieved for a dual-layered YVO_4_:Eu^3+^/YVO_4_:Dy^3+^ sample, exhibiting a maximum sensitivity of 3.6% K^−1^ at 640 K [102].

## 5. Sensing Applications: Recent Advances

Sensing applications of vanadates have been previously illustrated and discussed. This section provides a brief overview of significant developments in the field since 2023.

Vanadate materials—including binary oxides such as V_2_O_5_, ternary compounds like BiVO_4_, and various transition-metal vanadates (e.g., Zn–, Fe–, Pr–vanadates)—are semiconductors characterized by tunable electronic structure, high redox activity, and versatile surface chemistry. These properties render them highly effective for a range of chemical sensing applications, particularly in gas detection and electrochemical identification of complex analytes.

Vanadium pentoxide, for instance, exhibits a wide bandgap (2.3 eV), a robust chemical and thermal stability, a rich surface chemistry and a characteristic metal–insulator transition at 257 °C. As detailed in a comprehensive review of V_2_O_5_ gas sensors [105], these devices operate primarily through three mechanisms: gas adsorption, polaron hopping and direct chemical interaction. These mechanisms facilitate diverse applications in environmental monitoring, food safety, medical diagnostics and pharmaceutics. Recent literature [106] emphasizes that the high surface-to-volume ratio and inherent reactivity of V_2_O_5_ allow for the selective detection of biomolecules and other analytes at sub-ppm concentrations. Furthermore, its distinctive lamellar structure is highly conducive to doping, enabling direct modification of its structural and optical characteristics. Incorporating multiple Ln^3+^ elements can induce cooperative effects that enhance optical performance and expand functionality [107]. For instance, annealed films constituted by CeO_2_:V_2_O_5_ nanoparticles have recently demonstrated high sensitivity in thin film strain-gauge applications due to their optimized electrical and morphological characteristics [108].

Bismuth vanadate is an n-type semiconductor nanomaterial with a narrow bandgap (2.4 eV), making it highly active under visible light. While being a promising catalyst for rapid removal of pollutants from wastewater [109,110], BiVO_4_ has emerged as a potent biosensing platform, due to its unique electrochemical properties, high dispersibility, high photocatalytic efficiency, low toxicity, and biocompatibility. Recent developments highlight its efficacy in detecting various disease biomarkers with high sensitivity and specificity [111]. Additionally, morphology-tailored BiVO_4_ nanostructures have shown improved gas-sensing performance for environmental monitoring (e.g., NO_x_, H_2_S) by maximizing surface active sites and oxygen vacancies [112].

The zircon-type tetragonal structure of certain vanadates acts as a stable host lattice for dopant ions. Materials such as YVO_4_, GdVO_4_, and LuVO_4_ have emerged as premier materials for optical sensing due to their unique combination of structural stability and exceptional spectroscopic properties. Unlike other hosts, the vanadate group (VO_4_^3−^) is “self-activated”, absorbing ultraviolet (UV) light and efficiently transferring energy to Ln^3+^ dopants, thus significantly enhancing luminescence. These materials maintain structural integrity at high temperatures (melting points near 1800 °C) and are resistant to chemical degradation, making them ideal for sensing in harsh environments [113,114,115].

**Figure 11:**
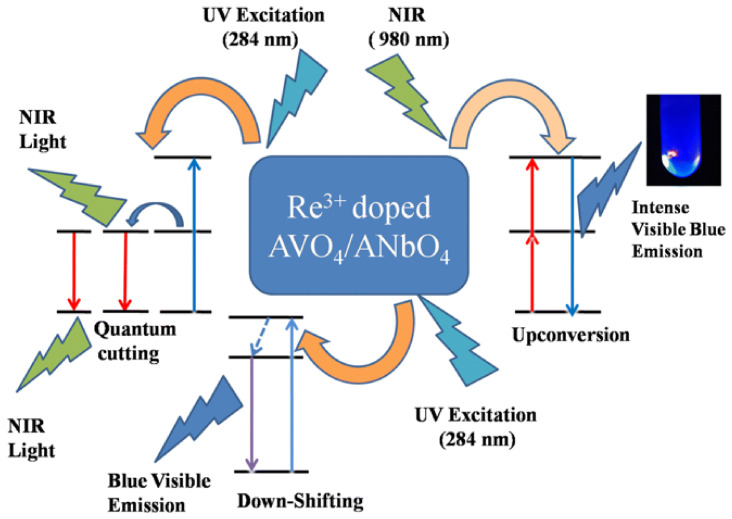
Schematic representation of the spectral conversion processes which may occur in RE-doped vanadate and niobate materials upon UV or NIR excitation. A = Y or Gd for vanadate, and A = Y, Gd, or La for niobate. Image reproduced from [11] under CC BY license.

Figure 11 schematically shows the luminescence processes in RE-doped AVO_4_ and ANbO_4_ phosphors (where A = Y, Gd, or La for niobate, and A = Y or Gd for vanadate) [9]. The very intense emission upon UV and NIR excitation may be exploited for producing high-security anti-counterfeiting inks. These ‘invisible’ inks remain covert under ambient light but emit intense fluorescence under UV or near-infrared (NIR) excitation. An advance in documents’ security is possible by integrating optical authentication provided by the ink’s fluorescence and biometric recognition through fingerprint patterns. Efficient detection of latent fingerprints, with low background interferences, was made possible by the RE ions’ capability of multi-color emission [116,117,118,119].

**Figure 12:**
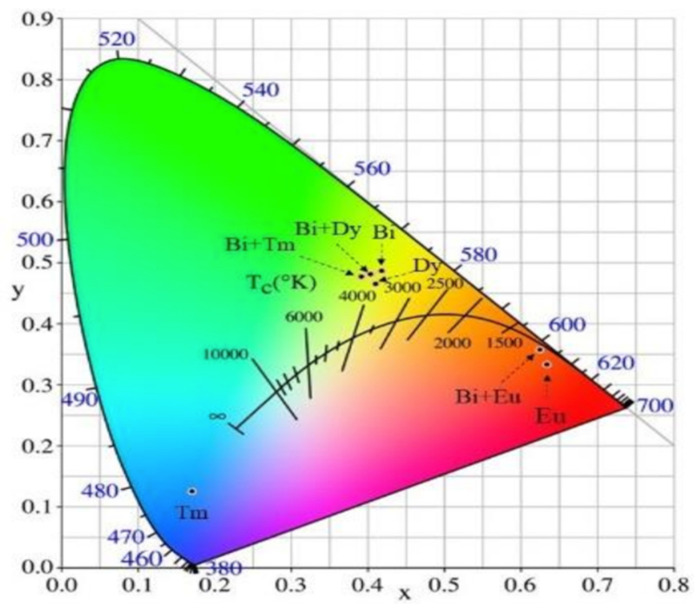
CIE color coordinates of gadolinium vanadate doped with heavy metal (Bi) and different rare-earth elements (Dy, Eu, Tm) or a combination of them (Bi+Dy, Bi+Eu, Bi+Tm). Reproduced from [117] under CC BY license.

For example, Ye et al. [117] developed various GdVO_4_:Ln^3+^ compounds through a dual sintering process at 1100 °C. A CIE color coordinate diagram for GdVO_4_ doped with trivalent Bi, Dy, Eu, Bi/Dy, Bi/Tm and Bi/Eu ions is shown in Figure 12. The GdVO_4_:Bi^3+^/Eu^3+^ formulation proved particularly resistant to background interference, enabling the resolution of latent fingerprint details on various surfaces and under high-humidity and -temperature conditions. Because these nanoparticles are significantly (1000–10,000 times) smaller than the width of a fingerprint ridge, they enable exceptional spatial resolution and superior adhesion. Recent studies on a set of nanocrystalline red-light-emitting Ca_8_ZnGd_1−x_Eu_x_(VO_4_)_7_ (x = 0.10–0.50 mol), synthesized via a non-hazardous and cost-effective solution-based calcination (SBC) route, have shown promising results [119]. XRD analysis confirmed that Eu^3+^ ions were diffused into the trigonal host matrix without causing structural deformation. The size of non-uniform agglomerated particles was evaluated around 61 nm. The chromaticity coordinates of the optimal sample (Ca_8_ZnGd_0.80_Eu_0.20_(VO_4_)_7_) and its high color purity of 97.7% indicate that this material stands at the forefront of both fluorescence-based forensic sensing and advanced optoelectronic devices. The recent review article by Ugemuge et al. [13] provides a critical evaluation of the performance of vanadate-based phosphors for optoelectronic devices. It bridges the gap between synthesis methods and functional outcomes by presenting real-world applications. This aspect of the review significantly enhances its sensibleness and relevance as a phosphor [13].

As a general trend, RE-doped vanadates have gained significant interest for luminescence thermometry and chemical biosensing. In most cases, the luminescence intensity ratio (LIR) technique is used. This ratiometric approach calculates absolute temperature by comparing the intensity of two distinct emission peaks (e.g., from Eu^3+^ or Nd^3+^), effectively neutralizing fluctuations in excitation laser power. Beyond LIR, thermal sensing can also be performed by monitoring temperature-dependent shifts in spectral line position and line bandwidth. Significant research over the last decade has demonstrated the efficacy of various nanostructures, including YVO_4_:Nd^3+^ [97] and La_3_Sc_2_Ga_3_O_12_:Cr^3+^/Nd^3+^, nanophosphors [120], YVO_4_: Ho^3+^/Yb^3+^ nanocrystals [121], GdVO_4_:Er^3+^/Yb^3+^ nanocrystalline powders [122], and (Y, Yb, Tm, Er)VO_4_ systems [123].

In a recent study, an RE-doped solid solution of yttrium phosphate–vanadate (YV_1–x_P_x_O_4_:Eu^3+^, Er^3+^) was proposed as a high-performance luminescent thermometer [124]. This material leverages the strong, broad charge-transfer absorption of the vanadate group to sensitize RE^3+^ ions. It facilitates multi-mode sensing by utilizing the LIR of Er^3+^ ^2^H_11/2_/^4^S_3/2_ emission, the dual-center LIR of integrated Er^3+^ and Eu^3+^ emission intensities, and the emission lifetime of Eu^3+^. This system enables very accurate temperature sensing across a wide temperature range, from room temperature to 873 K; inside that range, the P/V ratio (x) is adjustable to optimize performance for specific thermal windows.

While the LIR method is easy to be implemented and is robust against experimental or sample-related conditions, such as fluctuations in excitation intensity, sample geometry, or concentration of luminescent probes, concerns have been raised about its absolute precision. Vieira Perrella et al. [125] addressed this issue by synthesizing UV-excitable Eu^3+^-doped yttrium vanadate and phosphovanadate (Y(V,P)O_4_) particles. Their work clarified how the intrinsic spectral overlap between ^3^T_1,2_ → ^1^A_1_ VO_4_^3–^ transitions and Eu^3+^ transitions ^5^D_0_ → ^7^F_J_ impacts sensor reliability. Without a proper baseline correction, the broad band background overlap can result in a ten-fold increase in temperature uncertainty and a ∼60% underestimation of relative thermal sensitivity.

Vanadate compounds are increasingly used for the detection and remediation of persistent pollutants in aquatic ecosystems, often related to pharmaceutical residuals eliminated by humans and animals through their bodily waste. This issue has become crucial due to the disproportionate usage of antimicrobial agents in humans, animals and feed supplements. The photocatalytic activity of pure Ca_2_V_2_O_7_ has shown remarkable results under visible light irradiation [126]. Within 180 min, this material achieved 79.5% removal of safranin O (a red cationic dye used in histology and Gram-positive bacteria staining) and 80.6% removal of tetracycline hydrochloride (a yellow, crystalline, broad-spectrum antibiotic) [126]. In addition, Ca_2_V_2_O_7_ nanomaterial was effective for photocatalytic hydrogen generation.

REVO_4_ compounds are emerging as superior electrochemical probes for the precise quantification of various biological and pharmaceutical compounds. Sriram et al. [127] performed a comparative study of hydrothermally synthesized TVO_4_ (T = Ho, Y, Dy) for the simultaneous detection of two representative drugs, namely, nitrofurazone (an antiseptic drug for urinary tract infection) and roxarsone (an organo-arsenic drug, used in poultry as feed additive). The three RE vanadates were found to respond differently, and results demonstrated that DyVO_4_-based electrodes exhibited superior sensitivity, achieving exceptionally low detection limits (0.002 µM for nitrofurazone and 0.0009 µM for roxarsone). Similarly, orthovanadates (REVO_4_, where RE = Pr, Gd, and Sm) were evaluated for the detection of two antibiotics, i.e., furazolidone (FD) and metronidazole (MD), nitro-functional synthetic drugs that have been in use for over 30 years [128]. Using differential pulse voltammetry, the different vanadates were compared as electrode modifiers, and the SmVO_4_-modified glassy carbon electrode emerged as the most effective, characterized by the lowest charge-transfer resistance (R_ct_ = 56.82 Ω) and the largest electrochemical surface area (A = 0.11 cm^2^). This sensor not only provided the lowest limits of detection (0.0009 μM for FD and 0.0036 μM for MD individually, and 0.0015 μM and 0.0049 μM for simultaneous detection) but also showed excellent anti-interference, repeatability, and reproducibility. A recent comparative analysis of RE-doped molybdate, tungstate, and vanadate nanomaterials for the detection and photocatalytic degradation of nitrofurantoin (NFT), a persistent and toxic antibiotic contaminant, highlighted the versatility of these materials [129]. A series of NaDy(MoO_4_)_2_:Tb^3+^, NaDy(WO_4_)_2_:Tb^3+^, and Na_3_Dy(VO_4_)_2_:Tb^3+^ nanomaterials were synthesized via a hydrothermal method and systematically characterized. While the tungstate variant excelled in the photocatalytic degradation of NFT under UV light (96% degradation in 60 min), the vanadate variant exhibited the highest sensitivity for NFT detection, with a detection limit of 0.38 ppm [129]. Additionally, GdVO_4_ nanoparticles showed good photocatalytic and antimicrobial activity against bacteria *E. coli* and *Streptococcus mutans* and fungi *Aspergillus niger* and *Candida albicans* [130].

## 6. Conclusions

This paper provides a comprehensive overview of advances in synthesis strategies, luminescent properties and sensing applications of Ln^3+^ (Eu^3+^, Sm^3+^, Tm^3+^, Er^3+^, Ho^3+^, Tb^3+^, Nd^3+^, and Yb^3+^)-doped REVO_4_ (RE = Y, Gd, Lu, La) phosphors A detailed discussion is given regarding the preparation method, such as solid-state reactions, coprecipitation, hydrothermal/solvothermal, sol–gel, and microwave-assisted methods. The main attention has been focused on their structural and optical properties, with special reference to downconversion and upconversion luminescence, and the sensing application perspectives of these materials.

The undoped and Ln^3+^-doped REVO_4_ materials have emerged as versatile platforms for biodetection, fluorescence in vitro and in vivo bioimaging, and therapeutic applications including anti-aging, antibacterial, anticancer and antioxidant effects. Their performance is driven by advances in host materials such as GdVO_4_, YVO_4_, and LuVO_4_, alongside innovations in design, size, shape, co-doping and surface modification. As a result, undoped and Ln^3+^-doped REVO_4_ nanomaterials are gaining increasing importance in biomedical research, with rapid progress expected through continued developments in material synthesis, surface engineering and assay technologies.

REVO_4_ co-doped with Yb^3+^ or Nd^3+^, so-called upconverting materials, has broad sensing application potential, beyond biomedicine. It has been applied in luminescence imaging, cell labeling, magnetic resonance imaging, photodynamic therapy, chemotherapy, single-photon emission computed tomography, X-ray computed tomography, optical biosensing, biomarker identification, immunoassays, drug delivery, luminescence thermometry, solar energy conversion, fingerprint detection, photocatalysis, and related fields. In biomedical contexts, upconverting nanomaterials offer distinct advantages, including deeper tissue penetration, minimal autofluorescence background, reduced scattering and absorption, excellent photostability, and negligible photoblinking.

Looking ahead, the main research on undoped and Ln^3+^-doped REVO_4_ materials should be focused on advanced methods of synthesis, surface modification and polymer encapsulation. The exploration of multifunctional properties will broaden their applicability in emerging fields such as photonic devices, bioimaging, and environmental sensing. Shifting the excitation wavelength from 980 nm to safer biological windows, in particular around 800 nm, would give further advantages, increasing tissue penetration depth and reducing the tissue heating effect. In summary, these directions highlight the promising role of Ln^3+^-doped REVO_4_ in next-generation optical materials.

## Data Availability

This review article does not contain any original data. All data referenced in this article are publicly available from the sources cited in the references. No new datasets were generated or analyzed in this work.
